# Facile transformation of FeO/Fe_3_O_4_ core-shell nanocubes to Fe_3_O_4_ *via* magnetic stimulation

**DOI:** 10.1038/srep33295

**Published:** 2016-09-26

**Authors:** Aidin Lak, Dina Niculaes, George C. Anyfantis, Giovanni Bertoni, Markus J. Barthel, Sergio Marras, Marco Cassani, Simone Nitti, Athanassia Athanassiou, Cinzia Giannini, Teresa Pellegrino

**Affiliations:** 1 Istituto Italiano di Tecnologia, Via Morego 30, 16163 Genova, Italy; 2 IMEM-CNR, Parco Area delle Scienze 37/A, 43124 Parma, Italy; 3 Institute of Crystallography, National Research Council, via Amendola 122/O, Bari 70126 Italy

## Abstract

Here, we propose the use of magnetic hyperthermia as a means to trigger the oxidation of Fe_1−x_O/Fe_3−δ_O_4_ core-shell nanocubes to Fe_3−δ_O_4_ phase. As a first relevant consequence, the specific absorption rate (SAR) of the initial core-shell nanocubes doubles after exposure to 25 cycles of alternating magnetic field stimulation. The improved SAR value was attributed to a gradual transformation of the Fe_1−x_O core to Fe_3−δ_O_4_, as evidenced by structural analysis including high resolution electron microscopy and Rietveld analysis of X-ray diffraction patterns. The magnetically oxidized nanocubes, having large and coherent Fe_3−δ_O_4_ domains, reveal high saturation magnetization and behave superparamagnetically at room temperature. In comparison, the treatment of the same starting core-shell nanocubes by commonly used thermal annealing process renders a transformation to γ-Fe_2_O_3_. In contrast to other thermal annealing processes, the method here presented has the advantage of promoting the oxidation at a macroscopic temperature below 37 °C. Using this soft oxidation process, we demonstrate that biotin-functionalized core-shell nanocubes can undergo a mild self-oxidation transformation without losing their functional molecular binding activity.

Iron oxide nanoparticles are an indispensable candidate for varieties of nanoparticle-based therapeutics and diagnostics owing to their switchable magnetization, biocompatibility and biodegradation^1^,^2^,^3^. Magnetic hyperthermia is a novel non-invasive therapy, now under clinical trial on patients with brain or prostate tumors, that exploits magnetic nanoparticles as heat mediators to burn cancer mass^4^,^5^,^6^,^7^. The heat dissipation strongly depends on physico-chemical features of the particles. Up to now, there have been several studies aiming at the design of optimal heat mediators^8^,^9^,^10^. Recently, it was reported that anisotropic cubic-shape particles reveal a superior heating performance with respect to spherical ones, yet tightly relying on their structural and compositional properties^11^,^12^.

The synthesis of monodisperse iron oxide nanocubes is a great challenge and hardly attainable by any other method rather than high temperature colloidal syntheses. The iron pentacarbonyl and iron oleate are among the most frequently used precursors for the synthesis of iron oxide nanoparticles due to their particular decomposition profile that allows a distinctive separation between nucleation and growth steps, a vital criterion to obtain uniform nanocrystals^13^,^14^,^15^,^16^,^17^,^18^,^19^,^20^,^21^,^22^,^23^. Some of the developed synthetic procedures that make use of these precursors result in the formation of initial paramagnetic FeO (rock-salt (RS) structure) particles because of the reductive nature of the decomposition reaction^24^. After being exposed to ambient conditions, the outer particle surface transforms into Fe_3_O_4_ phase (inverse spinel (S) structure) and eventually core-shell structures having an antiferromagnetic core and a ferri(o)magnetic shell (AFM-FiM) are formed^25^. The core-shell particles, depending on the composition of the core and the shell, exhibit intriguing features such as exchange coupling between hard and soft magnets and exchange bias coupling which have raised lots of scientific and technological attentions^26^. For instance, the coupling between magnetically hard CoFe_2_O_4_ core and soft MnFe_2_O_4_ shell improves magnetization and magnetocrystalline anisotropy in these core-shell nanoparticles, making them a promising agent for magnetic hyperthermia^27^. It has also been shown that the formation of Fe_3_O_4_ (FiM) shell on CoO (AFM) particles, causing the exchange bias coupling, can modify and improve the magnetization of the resulting CoO/Fe_3_O_4_ core-shell system compared to the pristine CoO core^28^. In Co_x_Fe_1−x_O/Co_x_Fe_3−x_O_4_ (AFM-FiM) core-shell nanoparticles, the exchange bias and the coercive field were tuned by varying the dimension of the AFM core^29^. However, in the case of FeO/Fe_3_O_4_ particles, the presence of an AFM core significantly lowers the overall magnetization compared to single-phase Fe_3_O_4_ nanoparticles^30^. Hitherto, a way to improve magnetization and structure of these core-shell particles is based on post synthesis annealing at elevated temperatures^31^,^32^,^33^. However, the major drawback of such a harsh oxidation method is the destabilization and irreversible agglomeration of the particles, as the magnetic domain enlarges (*i.e.* for nanoparticles at the interface between superparamagnetic and ferromagnetic and for ferromagnetic nanoparticles) and the inter-particle dipole-dipole interactions, after annealing, prevail. Also, these treatments are usually performed in non-hydrolytic high boiling point solvents and the thin shell of surfactants at the surface of the nanocrystals is not capable of preventing an irreversible particle aggregation. To this end, it is important to find a milder oxidation method that triggers the transformation of the core/shell nanoparticles to a single magnetic phase while preserving their colloidal stability and protecting surface bioactive ligands.

Here, by exploiting magnetic hyperthermia (MH), a set up commonly employed to measure the heating ability of nanoparticles, we demonstrate the unprecedented potential of transforming Fe_1−x_O/Fe_3−δ_O_4_ core-shell nanocubes into Fe_3−δ_O_4_ phase, while maintaining the macroscopic temperature below the body temperature. This mild oxidation method boosts particle hyperthermia performance, while concomitantly preserving shape and colloidal stability of the nanocubes. Having employed several structural and magnetic analysis techniques, we unravel the gradual evolution of magnetization, crystal structure, and phase composition occurring under the magnetically stimulated oxidation in these core-shell nanocubes, accounting for the improved heat performance.

## Results

**Figure 1:**
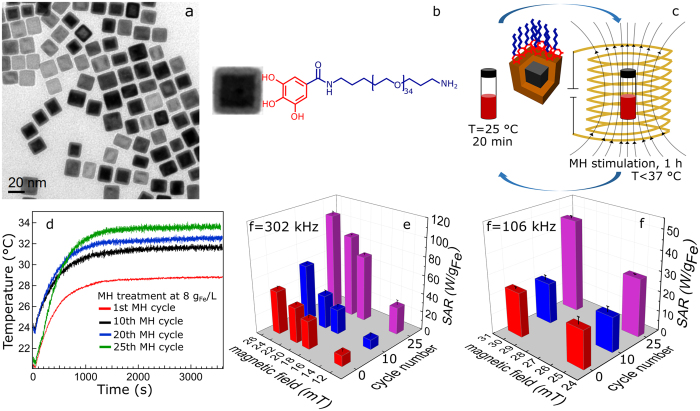
Magnetic stimulation treatment and SAR analysis. (**a**) Typical low resolution TEM micrograph of as-synthesized core-shell nanocubes, (**b**) scheme of gallic-PEG-NH_2_ coated core-shell nanocube, (**c**) schematic representation of a MH treatment cycle, (**d**) heating profiles of PEG coated nanocubes *vs.* MH cycle number (particles concentration fixed at 8 g_Fe_/L), (**e,f**) the temporal evolution of the SAR over 25 MH treatment cycles as a function of magnetic fields measured at two fixed frequencies of 302 (**e**) and 106 (**f**) kHz, respectively.

The Fe_1−x_O/Fe_3−δ_O_4_ nanocubes, with an average edge length of 17 ± 2 nm, were synthesized by decomposition of iron pentacarbonyl Fe(CO)_5_ in a mixture of 1-octadecene, oleic acid, and sodium oleate. A typical TEM image is shown in Fig. 1a (particle size histogram is plotted in Fig. S1a of the Supplementary Information (SI)). The nanocubes were transferred into water by exchanging their original surfactants with a gallic acid derivate of the α-ω-diaminopropyl-poly(ethylene glycol) polymer (Mn = 1500 g/mol) (Gallic-PEG-NH_2_, Fig. 1b) synthesized by a one-step reaction with a degree of functionalization of 30%, as determined by ^1^H NMR (Supplementary Fig. S2). The water transfer approach allows attaining single-core particles as discerned from TEM studies (Supplementary Fig. S1b).

### Magnetic hyperthermia stimulation and SAR analysis

In a typical MH treatment cycle, the PEGylated nanocubes in water were sequentially exposed to 1 h alternating magnetic fields at 330 kHz and 17 mT followed by 20 min rest time at ambient conditions and this cycle was repeated for a certain number of times as schematically shown in Fig. 1c. For a given frequency, SAR values were recorded as a function of magnetic fields on the sample of the untreated nanocubes and after a certain number of cycles, usually 10 and 25 MH cycles (Fig. 1e,f). Notably, at all field values, higher SAR values were recorded for the samples that underwent the magnetic stimulation. By increasing the frequency from 106 to 302 kHz, as expected, the SAR values rise. For instance, the SAR value at 302 KHz and 25 mT increases from 47 W/g_Fe_ to 106 W/g_Fe_ after 25 MH treatment cycles, indicating the change in structural and magnetic properties of the particles. Although the highest temperature recorded during the treatment never exceeded 34 °C, a gradual increase of the initial slope of the temperature *vs.* time curve and the maximum temperature reached at the end of the cycle was always registered as more treatment cycles were applied (Fig. 1d).

To furthermore support the magnetically stimulated phase transformation hypothesis, rather than a bulk temperature effect *per se* (the macroscopic temperature raised solely for few degrees during the full MH treatment and never exceeding 34 °C), in a control experiment, the initial core-shell nanocubes were heated in water bath for 25 h (*i.e.* corresponding to 25 MH treatment cycles) applying the same heating profile as recorded in the MH treatment. For the sample heated up in the water bath, the SAR value measured at 302 kHz and 25 mT was only 49 W/g_Fe_, showing a marginal increase compared to the SAR of the initial sample (47 W/g_Fe_). Upon applying alternating magnetic fields, the particles go through multiple magnetization-demagnetization hysteresis loops, resulting in the conversion of magnetic to heat energy. It is known from previous studies that the temperature at the surface proximity of nanoparticles is substantially higher than the macroscopic temperature detected by the optic fiber sensor in the solution. Apparently, these hot spots can accelerate the oxidation and structural change in the core-shell nanocubes and consequently improve their heat performance^34^,^35^. It is also worth mentioning that the final SAR values recorded on the nanocubes after 25 cycles of MH treatment are higher than SAR values, measured in a comparable field and frequency, reported for iron oxide nanoparticles and nanoclusters obtained from other direct synthesis methods^36^,^37^.

### High resolution electron microscopy characterization

**Figure 2:**
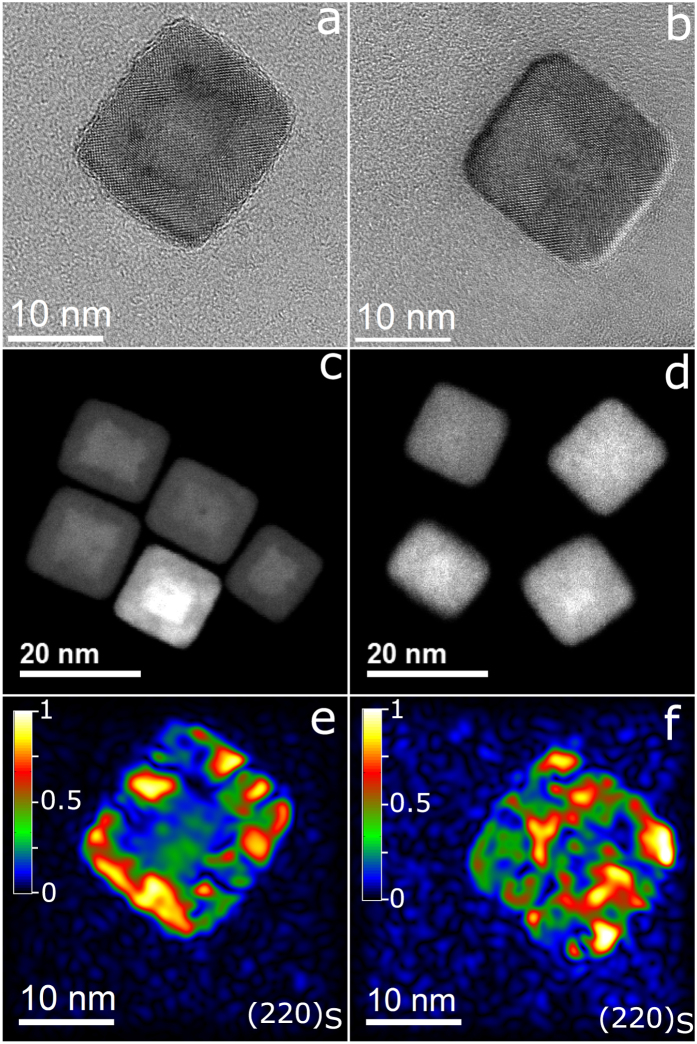
High resolution electron microscopy characterization. (**a,b**) HRTEM, (**c,d**) STEM micrographs of initial core-shell nanocubes and 25 MH cycles treated nanocubes, and (**e,f**) amplitude maps of relative intensity of {220}_S_ spinel-only fringe of initial core-shell and 25 MH cycles treated nanocubes, respectively, obtained from the GPA analysis. The {220}_S_ corresponds to the Fe_3_O_4_ phase. The [001] zone-axis lies parallel to the electron beam.

The core-shell structure of the initial nanocubes can easily be discerned by looking at HRTEM, and it is even more evident in the STEM images (Fig. 2a,c). On the same nanocube treated for 25 MH cycles, a more homogenous and ordered crystal structure was detected with no trace of the initial core-shell structure (Fig. 2b,d). To gain more information about the phase structure of the nanocubes, geometric phase analysis (GPA)^38^ was performed (see the SI). The amplitude map of {220}_S_, a fringe only ascribable to the Fe_3−δ_O_4_, (Fig. 2e) shows a significantly higher magnitude in the outer layers than in the core of the initial nanocubes. In the amplitude map, a higher intensity corresponds to a higher occurrence of the corresponding plane. Instead, the amplitude map of {220}_S_ fringe of 25 MH cycles treated nanocubes reveals high magnitude on both the shell and the core of the particles, thus implying the growth of the Fe_3−δ_O_4_ phase towards the core (Fig. 2f). Based on the discontinuities observed in the amplitude map of the {220}_S_ fringe, it appears that the Fe_3−δ_O_4_ phase nucleates as small subdomains on the shell of nanocubes which grow larger during the oxidation. This island-like nucleation and growth has previously been observed in iron oxide core-shell nanoparticles^25^,^32^. Compared to the amplitude map of {400}_S_-{200}_RS_ of the initial nanocubes which is mainly brighter in the center (Supplementary Fig. S3a), the amplitude map of {400}_S_ fringe after the MH treatment, shows a high magnitude throughout the whole nanocube (Supplementary Fig. S3b).

### Field and temperature dependence of magnetization

**Figure 3:**
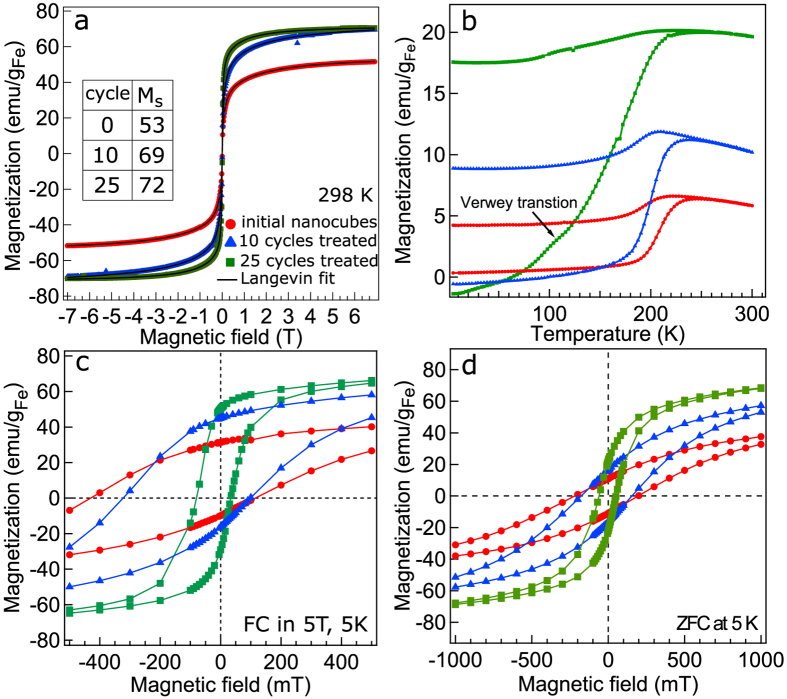
Field and temperature dependent magnetization. (**a**) M-H magnetization loops measured at room temperature (symbols) and the best fits (solid lines), (**b**) temperature dependent zero-field-cooled (ZFC) and field-cooled (FC) magnetizations measured at 10 mT, (**c**) FC cooled at 5 T and (**d**) ZFC hysteresis loops of immobile particles recorded at 5 K for the initial core-shell nanocubes (red symbols), 10 (blue symbols) and 25 (green symbols) MH cycles treated nanocubes.

Since a change in the structural features of the nanocubes is reflected in their magnetic properties, the magnetization hysteresis loops were compared before and after the MH treatment. Interestingly, the superparamagnetic behavior of the initial core-shell nanocubes was not affected by the MH treatment, and samples after 10 and 25 MH cycles revealed zero coercive fields at 298 K as the initial core-shell nanocubes did. As an indicator of the change in the structure, it is important to trace the saturation magnetization (*M*_*s*_). At 298 K, *M*_*s*_ increases from 53 emu/g_Fe_ for the initial nanocubes to 69 emu/g_Fe_ and eventually to 72 emu/g_Fe_ after 10 and 25 MH treatment cycles, respectively (Fig. 3a). At 5 K, *M*_*s*_ value of 25 MH treated nanocubes is 94 emu/g_Fe_ (corresponding to 73 emu/g_Fe3O4_), deviating from the bulk magnetite value^39^ (Table 1). This deviation is mainly due to a tiny remained fraction of FeO after full MH treatment as discussed later. The hysteresis curves were reconstructed using a discrete form of the Langevin function to acquire *M*_*s*_ and particle magnetic moment (*m*) distribution (see SI)^40^. It can be realized that *m* distribution and the corresponding magnetic domain size (*d*_*m*_) (given in Table 1) grow slightly during the first 10 cycles, and then increase significantly in the last 15 cycles. Also, the magnetic moment distribution becomes narrower as the Fe_3−δ_O_4_ fraction enlarges (Supplementary Fig. S4). Conversely, *M*_*s*_ does reveal a rather significant rise within the first 10 cycles. These results demonstrate that *M_s_* and *d*_*m*_ do not always show a linear correlation particularly in such complex core-shell nanocubes.

By looking at the temperature dependent zero-field-cooled (ZFC) and field-cooled (FC) magnetizations (Fig. 3b) it can be seen that the magnetization of initial core-shell and that of 10 cycles treated nanocubes rises slightly up to the Néel temperature (*T*_*N*_) of FeO at 180 K. A huge rise seen at this temperature is due to the transition from antiferromagnetic to paramagnetic spin configuration in FeO. Differently, on the nanocubes exposed to a full 25 MH treatment cycles, a clear kink at 110 K in the ZFC curve is seen which can be attributed to the Verwey transition in magnetite^25^,^31^. A long-range crystal structure ordering in 25 cycles treated nanocubes can be accounted for this observation. Prominently, the Verwey transition has not been observed in core-shell nanocubes which were annealed at 150 °C^32^, analogous to the here studied nanocubes after thermal annealing at 130 °C (Supplementary Fig. S10b). This suggests that in the present Fe_1−x_O/Fe_3−δ_O_4_ nanocubes the magnetic heating triggers the phase transformation *via* different pathways than plain high temperature annealing. The superparamagnetic blocking temperatures (*T*_*b*_) were estimated from the maximum peak in the ZFC curve (refer to Table 1). As the particles undergo more cycles of MH treatment, *T*_*b*_ decreases. Also, the magnetocrystalline anisotropy constant (*K*_*e*_), estimated using the Néel-Brownian relaxation equation (see SI), shows a decreasing trend as the Fe_3−δ_O_4_ magnetic domain becomes larger and more coherent upon applying MH cycles (see Fig. S5 for ZFC curves measured at other field amplitudes). The *K*_*e*_ values here reported match the mostly recognized ones for magnetite *i.e.* 11–13 kJ/m^3^ (Table 1)^41^.

The spin configuration in Fe_1−x_O is paramagnetic at room temperature and upon cooling below its *T*_*N*_, the spins orient antiferromagnetically. However, part of the spins at the Fe_1−x_O/Fe_3−δ_O_4_ interface remains uncompensated which pin the Fe_3−δ_O_4_ spins towards the cooling field. These AFM-FiM interfaces generate large exchange bias fields *H*_*EB*_, identified by shifted hysteresis loops in the opposite direction of the applied fields^42^. The measured FC magnetization curves performed on particles cooled to 5 K in 5 T, show that as the particles are progressively oxidized, both horizontal and vertical shifts in the FC loops are reduced (Fig. 3c). This is an indication of the shrinkage of Fe_1−x_O core and AFM-FiM interfacial spins. *H*_*EB*_ lowered significantly after 25 MH treatment cycles, not yet entirely vanished (Table 1). A similar trend was observed in Fe_3_O_4_ films and was linked to the presence of antiphase boundaries (APBs) at the interface of growing magnetic domains^43^,^44^,^45^,^46^. The ever existing *H*_*EB*_ field was detected in iron oxide nanocubes oxidized post synthesis up to 48 h as well as in single-phase Fe_3_O_4_ and γ-Fe_2_O_3_ nanocrystals^33^,^36^,^39^,^47^. It appears that on the nanocubes here studied, the exchange coupling plausibly has a structural origin. ZFC hysteresis loops reveal no asymmetric behavior. Besides, *H*_*c*_ reduces as the spinel domains grow larger, matching the reduction of *K*_*e*_.

### Structural and compositional analysis

**Figure 4:**
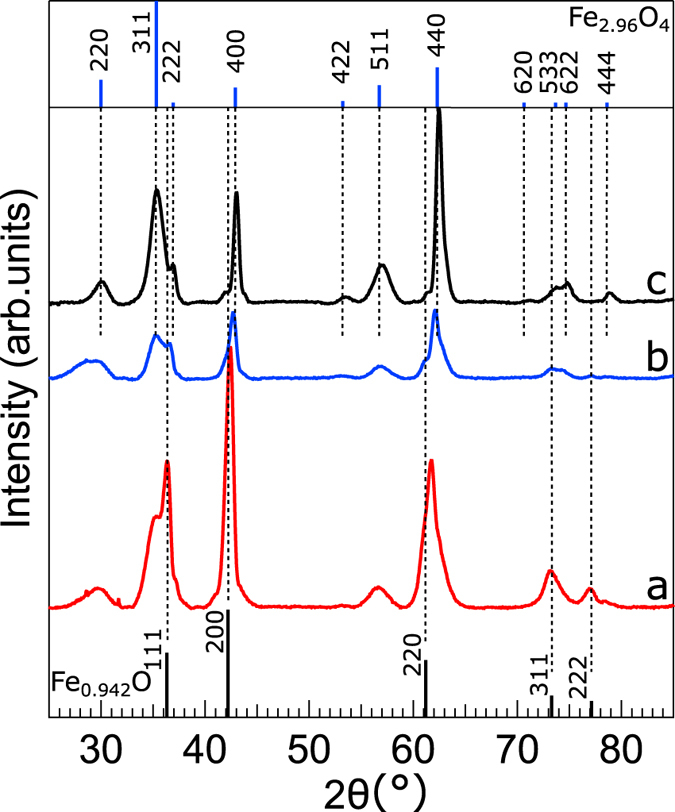
X-ray diffraction analysis. Powder X-ray diffraction patterns of (**a**) initial core-shell, (**b**) 10, and (**c**) 25 MH cycles treated nanocubes. Theoretical diffraction lines of Fe_0.942_O (ICSD: 98-002-4696) and Fe_2.96_O_4_ (ICSD: 98-008-2443) are plotted at the bottom and upper panels, respectively. The dashed lines are guides for the eye.

The powder X-ray diffraction (XRD) and quantitative Rietveld analysis were used to gain information about the evolution of phase composition, lattice constant, and crystallite size of the nanocubes before and after the MH treatment. The XRD pattern of the initial core-shell nanocubes unambiguously shows the reflections ascribed to both Fe_1−x_O and Fe_3−δ_O_4_ phases having rock-salt (RS) and inverse spinel (S) crystal structure, respectively. The peak positions of Fe_1−x_O phase match with Fe_0.942_O (ICSD: 98-002-4696). After 10 and 25 MH cycles, the XRD patterns of the nanocubes show a concomitant drop in the intensities of (111)_RS_, (200)_RS_ and (220)_RS_ reflections of Fe_1−x_O and rise in the intensities of Fe_3−δ_O_4_ reflections (Fig. 4). The peak positions of 25 MH cycles treated nanocubes coincide perfectly with Fe_2.96_O_4_ (ICSD: 98-008-2443). The reconstructed patterns by Rietveld method, plotted on the experimental ones (Supplementary Fig. S6), revealed that the fraction of Fe_1−x_O declines gradually and after 25 MH cycles becomes significantly small (not reliably quantifiable by the Rietveld method). Conversely, the fraction of Fe_3−δ_O_4_ increases and eventually becomes dominant after 25 MH cycles (Table 1). Initially we have tried to take both Fe_1−x_O and Fe_3−δ_O_4_ phases into Rietveld analysis of the 25 MH cycles treated particles, but the fit quality was not satisfying. These trials hinted that there is still a small portion of Fe_1−x_O (5–10%) in the fully treated nanocubes. This together with the nominal atom occupancies assumed in the refinements (Table S1) are reflected in the difference in the relative peak intensities between the measured and simulated patterns (Supplementary Fig. S6c). The lattice constant of Fe_1−x_O and Fe_3−δ_O_4_ for the initial nanocubes was found to be 4.27 Å and 8.46 Å, corresponding to 0.8% contraction and expansion in the unit cell, respectively, if compared to the bulk values given for wüstite and magnetite (*i.e.* 4.30 Å and Å 8.39 Å respectively)^48^,^49^. Strikingly, the Fe_1−x_O lattice constant further decreases to 4.24 Å as its domain shrinks, presumably due to an increasing pressure by the growing Fe_3−δ_O_4_ subdomains. Accordingly, it can be discerned that the Fe_1−x_O core is under a huge pressure, even up to few GPa as shown in studies by McCammon^49^ and Hazen^50^. The lattice constant of Fe_3−δ_O_4_ reaches 8.39 Å after 25 MH stimulation cycles, matching perfectly the magnetite bulk value^48^.

The crystallite size of Fe_1−x_O and Fe_3−δ_O_4_ phases was estimated using the Scherrer equation (see the SI). The Fe_1−x_O crystallite size of (200)_RS_ line changes from 105 Å for the initial particles to 73 Å after 10 MH cycles. The Fe_3−δ_O_4_ crystallite size of (400)_S_ line rises steadily from 65 Å to 149 Å after 25 MH cycles. This indicates that the remaining domain of Fe_1−x_O is 21 Å, corresponding to a very small fraction of a 170 Å nanocube. On the contrary, the size of (220)_S_ increases marginally (Table 1). A similar retarded growing trend was observed, though less profound, for (440)_S_ reflection. This anisotropic line broadening is plausibly due to varying density of crystal defects (*e.g.* APBs) and stress along different crystallographic directions, in the case of (220)_S_ plane chiefly originated from the nucleation and growth of the spinel subdomains on the tetrahedral Fe^3+^ sublattice^51^. The formation of large spinel crystallite indicates a long-range crystal ordering in the fully treated particles, matching the appearance of the Verwey transition.

Principally, Fe_1−x_O is an oxygen-sufficient phase which hampers the diffusion of oxygen through its lattice. Therefore, the most plausible oxidation mechanism in these nanocubes is the concomitant absorption of O^2−^ into the shell and the diffusion of Fe^2+^ outwards the core and its subsequent oxidation to Fe^3+^ at the interface. The undistorted rotational ordering of the oxygen face-centered cubic (fcc) sublattice deduced from the GPA analysis (Supplementary Fig. S7) indicates a topotactical growth of the Fe_3−δ_O_4_ domains on the Fe_1−x_O core. The coalescence of the growing Fe_3−δ_O_4_ subdomains, typically shifted by ¼ a_0_[110], ½ a_0_[100] or/and rotated by 90°, causes the nucleation of the aforementioned APBs^43^,^44^,^52^,^53^,^54^. The formation of APBs in iron oxide nanocubes has been demonstrated and discussed by Wetterskog *et al*.^32^. Here, a similar behavior was observed that is the APBs do continue to persist even in the 25 cycles magnetically oxidized Fe_3−δ_O_4_ nanocubes (Supplementary Figs S8 and S9). The APBs separate the Fe_3−δ_O_4_ domains, being responsible for the retarded growth of the particle crystallite size along (220)_S_ diffraction line.

To compare the effect of conventional thermal annealing with the MH oxidation treatment, the same pristine nanocubes used for the MH study, before being transferred in water, were heated at 130 °C for 5 h (denoted by TA-130, the detailed procedure is described in the SI). Interestingly, a lattice constant of 8.35 Å was found for the TA-130 nanocubes by XRD and Rietveld analysis (Supplementary Fig. S10a), matching with the values given in the literature for γ-Fe_2_O_3_ nanoparticles^48^. Note that the particle crystallite size along (220)_S_ diffraction line rises to 61 Å, slightly larger than the value obtained for the 25 MH cycles treated nanocubes. Moreover, no shoulder at 110 K linked to the Verwey transition was identified in the ZFC magnetization curve (Supplementary Fig. S10b). This implies that the core-shell nanocubes are transformed to γ-Fe_2_O_3_ by the thermal annealing. Also looking at the M-H curve, the TA-130 nanocubes reveal 10% higher *M*_*s*_ value at 298 K compared with the magnetically treated ones (Supplementary Fig. S10c) and *d*_*m*_ of the TA-130 nanocubes was estimated 117 Å, slightly larger than the one obtained for the MH treated ones (Table 1). Notably, *H*_*EB*_ has not entirely disappeared in the annealed TA-130 nanocubes as identified by the shifted FC magnetization curve (Supplementary Fig. S10e). The presence of the APBs even after thermal annealing could be a plausible reason for this observation. It has been reported that to obtain a completely APB-free structure a much stronger diffusive motion is required which occurs at temperatures much higher than 130 °C^55^. The PEG coated TA-130 nanocubes gave SAR values 15% higher than the 25 MH cycles treated samples. However, the PEGylated TA-130 nanocubes show a broader hydrodynamic size distribution, having a fraction of 100 nm clusters (Supplementary Fig. S11a). The presence of aggregates formed as a result of harsh thermal annealing was confirmed by TEM studies (Supplementary Fig. S11b). Additionally, the yield of the water transfer TA-130 nanocubes was not as quantitative as the MH treated ones, revealing an advantageous feature of the proposed mild oxidation process. Owing to the crucial importance of producing single-core particles for *in vivo* applications, the thermally annealed nanocubes indeed have limited applications.

### Bio-activity and colloidal stability

The main distinctive feature of the here proposed magnetically triggered oxidation compared to typical thermal annealing processes^31^,^32^,^56^,^57^,^58^ is that the oxidation of the core-shell Fe_1−x_O/Fe_3−δ_O_4_ nanocubes to Fe_3−δ_O_4_ occurs under mild conditions (below 37 °C as opposed to the 130 °C of the thermal annealing). The MH treatment, being a process that is performed on core-shell nanocubes transferred in water when the inter-particle interactions are virtually absent (due to the antiferromagnetic-ferrimagnetic structure), enables quantitative water transfer of well soluble and single core-shell particles with excellent colloidal stability. The shape and single-core nature of the nanocubes indeed remain well preserved after 25 MH treatment cycles (Supplementary Fig. S12).

**Figure 5:**
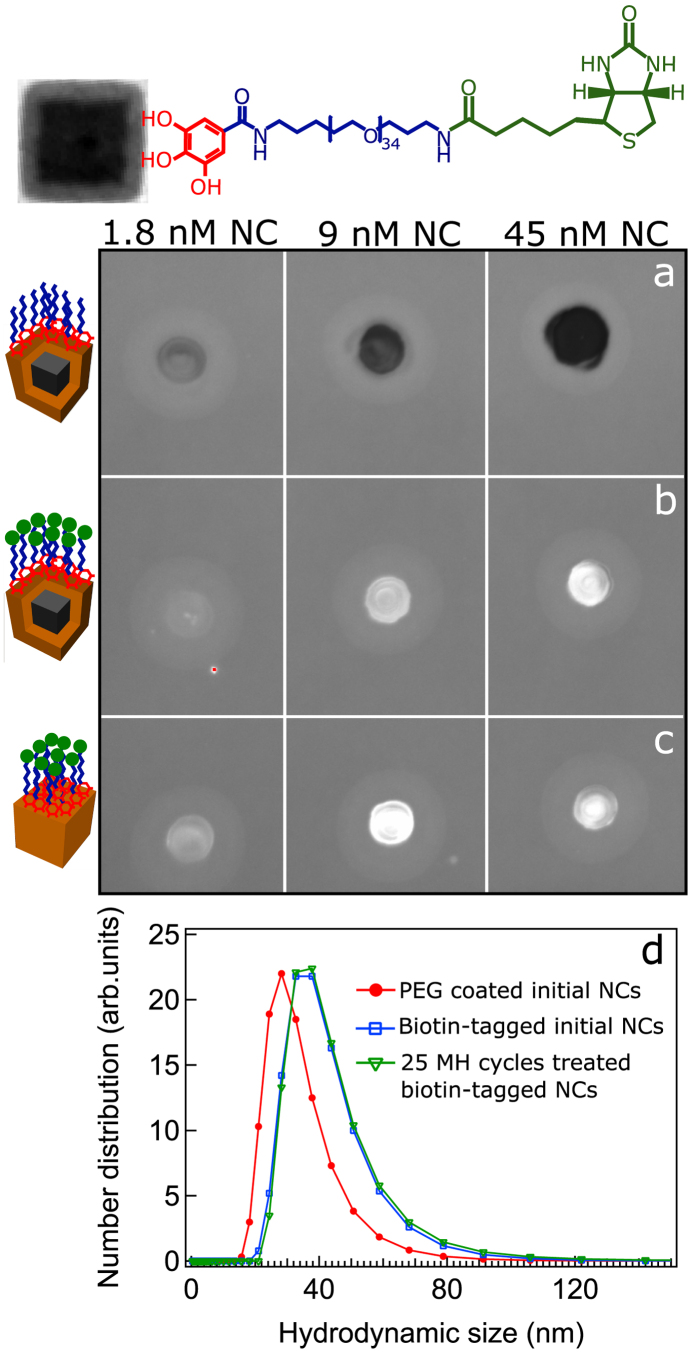
Functional and colloidal stability assays of biotin-modified nanocubes. Optical images taken at excitation wavelength of 488 nm from the dot blot nitrocellulose membrane of PEGylated core-shell nanocubes (**a**), biotin-functionalized nanocubes (**b**) prior and (**c**) after 25 MH treatment cycles spotted at three different particle concentrations after being treated with FITC-streptavidin. The fluorescence signal indicates the presence of streptavidin bound to the biotin on the nanocubes before and after the MH treatment. (**d**) Hydrodynamic size distribution (number weighted) of PEG coated (red line) and biotin-tagged nanocubes prior (blue line) and after (green line) 25 MH treatment cycles. On biotin-nanocubes no change in hydrodynamic size is observed before and after the MH treatment.

Moreover, the mild temperature of the MH treatment also implies that biomolecules attached to the nanocube surface may preserve their functionality (*i.e.* targeting properties). To prove this point, the amino terminated moieties of PEG molecules on the nanocube surface were reacted with NHS carboxyl-activated biotin molecules (Fig. 5). After biotin functionalization, the hydrodynamic diameter of the nanocubes increases from 30 nm to 40 nm. Given the mono-modal size distribution and the absence of other peaks, the single-core nature of the biotin functionalized nanocubes was retained. In addition, the particle surface potential drops from 15 mV to 8 mV after biotin functionalization, indicating a change in the particle surface chemistry after biotin attachment.

The colloidal stability of biotin modified nanocubes after exposure to 25 MH treatment cycles obviously remained unaffected (Fig. 5d). The dot blot assay was performed to evaluate the binding affinity of the biotin on the particle surface towards the FITC-streptavidin (Fig. 5a–c). The nanocubes, prior and after having being exposed to the MH treatment, were spotted on the nitrocellulose membrane. After addition of the FITC-streptavidin solution, the fluorescence signals were detected on the spots, indicating that surface biotin was still able to bind streptavidin even if the nanocubes were previously exposed to the MH treatment. The spots emit a fluorescence signal even at low nanocubes concentration (1.8 nM) which indicates a high density of biotin molecules attached per nanocube. On the contrary, the PEGylated nanocubes bearing no biotin molecules did not emit any fluorescence signal.

## Discussion

In summary, we have presented a novel self-oxidation approach whereby the heat dissipated by the particles *via* hysteresis and relaxation processes is exploited to transform the Fe_1−x_O/Fe_3−δ_O_4_ core-shell nanocubes to a major Fe_3−δ_O_4_ phase. The structural and magnetic characterizations confirmed the occurrence of magnetically triggered phase transformation, being responsible for the doubling of the SAR values. Moreover, since this process occurs in mild conditions, we have demonstrated the possibility of conducting this treatment on nanocubes functionalized with biotin without altering its binding affinity towards streptavidin and concomitantly preserving the colloidal stability of the MH treated nanocubes. The magnetically self-oxidized nanocubes possess all crucial physico-chemical features for being efficient heat mediators for *in vivo* cancer treatment by magnetic hyperthermia. These highly monodisperse and non-interacting core-shell nanocubes are potentially useful as heat mediators that upon magnetic hyperthermia stimulation at the tumor site may gradually improve their heating performance in a self-regulatory manner.

## Methods

### Synthesis of nanocubes

In a typical synthesis to obtain 17 nm nanocubes, 5 mL (0.015 mmol) ODE, 1.98 g (7 mmol) OLAC and 0.91 g (3 mmol) sodium oleate were added into a 50 mL three neck glass flask. Afterwards, the mixture was degassed at 110 °C for ≈1 h until no further bubbling was observed. The mixture was cooled to 60 °C and then 400 μL (3 mmol) Fe(CO)_5 _dissolved in 1 mL ODE, freshly prepared in glove box, was injected into the flask. The iron precursor injection and the growth steps were carried out under nitrogen flow. The resultant yellowish mixture was heated up to 320 °C within 25 min. The mixture firstly turned to dark orange and eventually after 1 h to black, indicating the nucleation of initial crystallites. From the time of the nucleation, the mixture was kept at this temperature for additional 90 min. After cooling the obtained black suspension to 80 °C, 40 mL chloroform was added to efficiently disperse the particles and then it was immediately cooled to room temperature (RT). The particles were precipitated by adding a 1:2 methanol/acetone solution and collected by centrifugation at 6000 rpm for 10 minutes. The precipitated particles were redissolved in fresh chloroform and the centrifuging steps were repeated five times to obtain thoroughly clean particles. Finally, the particles were readily dispersed in 40 mL chloroform and kept in glove box.

### PEG functionalization of nanocubes

To transfer the particles into water, their original oleate surfactant ligands were exchanged with the synthesized Gallic-PEG-NH2 molecules. Typically, 10 mL solution of nanocubes in chloroform at an iron concentration of 2 g/L was mixed with 15.6 mL (0.05 M) solution of Gallic-PEG-NH2 in chloroform and then 10 v% triethylamine was added. The mixture was stirred vigorously overnight and afterwards the solvent was evaporated off by purging nitrogen. Next, the waxy mixture of particles was dispersed in 20 ml Milli-Q distilled water by intensive nitrogen purging and vigorous shaking. After removing all volatile solvents, the particle suspension was transferred into RC dialysis membrane (Spectra/Por) with a cutoff of 25 kDa. The dialysis was carried out for three days to remove excessive polymers. Further cleaning of the particles was performed by centrifuging on Amicon filter tubes with 100 kDa cutoff at RT, 2500 rpm and for 25 minutes. This step was repeated five times and at the end the sample volume was reduced to 5 mL. By applying such extensive cleaning process, we can make sure that the obtained particles are free from excess polymer molecules. Typical TEM image of PEG coated nanocubes are seen in Fig. S1b.

### Biotin functionalization of nanocubes

The EDC-NHS coupling chemistry was utilized to bind biotin on the nanocube surface. First, the amino-PEG coated nanocubes were dispersed in MES buffer (0.01 M, pH 5.5) at a final particle concentration of 0.18 μM. A 180 μM solution of NHS-biotin was prepared by dissolving 0.001 g NHS-activated biotin firstly in 200 μL DMSO and then 29.8 mL MES buffer. To 1 mL nanocube MES solution, 1 mL biotin solution (180 μM) was added such that the molar ratio of biotin/NC is 1000. Subsequently, 1 mL EDC MES buffer solution (0.45 M) was poured into the mixture such that the molar ratio of EDC/NC is 250000. The mixture was stirred vigorously at RT overnight. Afterwards, unbound and excessive biotin molecules were removed by filtering the suspension through Amicon 100 kDa filter tube. The cleaning process was repeated five times. At the last filtering step, the particle suspension was concentrated to the desired iron concentration for further analysis and magnetic hyperthermia treatment.

### Magnetic hyperthermia treatment of aqueous nanocubes solution

Magnetic hyperthermia (MH) treatment was performed on aqueous solution of both PEG coated and biotin-functionalized nanocubes utilizing magneTherm setup (NanoTherics) operating at 330 kHz and 17 mT. The experiments were performed on 300 μL suspension having iron concentrations in the range between 4 to 10 g_Fe_/L. Each magnetic treatment lasts one hour and between each MH treatment cycle, the field was turned off and the sample was left to cool down to RT (typically it took about 20 minutes to cool the sample to RT).

### SAR measurements

The calorimetric measurements to quantify the specific absorption rate (SAR) value of the nanocubes were conducted using Nanoscale Biomagnetics instrument operating over a broad range of fields and frequencies. The SAR was calculated using the corrected slope method given by^59^,^60^


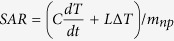


in which *C* is the dispersing medium heat capacity in J/K, *L* is the linear-loss parameter in W/*K*, ∆*T* is the temperature difference between the sample and baseline (the solution temperature before switching off the field) and *m*_*np*_ is the total particle mass. This method provides more accurate SAR values because it takes into account the heat dissipation from the medium to the environment.

### Field and temperature dependent magnetization curves

Field dependent static magnetic measurements were carried out by employing an ever cooled Magnetic Property Measurement System (*MPMS-XL*, Quantum Design) on immobile nanocubes. The immobile samples were prepared by mixing 50 μL of nanocube water solution at an iron concentration of 1–2 g/L with 60 mg gypsum in the designated polycarbonate capsules and let it to dry thoroughly. The zero-field-cooled (ZFC) and field-cooled (FC) temperature dependent magnetization measurements were performed on samples prepared in the same way in the cooling field of 10 mT. The FC M-H hysteresis loops were recorded after cooling the samples from RT to 5 K in 5 T magnetic fields. The residual magnetic field in the SQUID magnets was nulled using the designated low field Hall sensor prior to ZFC measurements. All the presented magnetization data are corrected with respect to the diamagnetic and paramagnetic contributions of water and gypsum using the automatic background subtraction routine. The curves were normalized to the iron concentration as obtained from the elemental analysis.

### Elemental analysis

The elemental analysis was performed using Inductively Coupled Plasma-Atomic Emission Spectroscopy (ICP-AES) instrument (Thermo Fisher, iCap 6000). Typically, 10–50 μL nanocube suspension was digested in 1 mL Aqua Regia in volumetric flask. Afterwards, the flask was filled up to the graduation mark with Milli-Q water and filtered through 0.2 μm membrane prior to the measurement.

### Powder X-ray diffraction

Powder X-ray diffraction (XRD) analysis was conducted on a Rigaku SmartLab diffractometer machine operating at 150 mA and 40 kV. The patterns were acquired in Bragg-Brentano configuration using D-tex Ultra 1D detector in the reflection mode. The samples were prepared by drop casting of concentrated particle suspensions on a zero diffraction silicon wafer.

### Transmission electron microscopy

Typical low resolution transmission electron microscopy (TEM) images were acquired using a JEOL JEM-1011 microscope operated at 100 kV. High resolution TEM (HRTEM) and annular dark field scanning TEM (STEM) imaging were carried out on a JEOL JEM-2200FS microscope equipped with a Schottky gun operated at 200 kV accelerating voltage. The samples were prepared by drying a drop of diluted particle suspensions on 400 mesh ultra-thin carbon coated TEM copper grids.

### Hydrodynamic and zeta potential

The particle hydrodynamic size distribution and zeta potential were measured utilizing Malvern Zetasizer operated in the 173° backscattered mode on highly diluted aqueous solution of nanocubes. The measurements were performed at 20 °C.

### Dot Blot assay

Dot Blot assay was carried out on pre-activated nitrocellulose membrane. Typically, 5 μL particle suspension was spotted on the membrane and left to dry thoroughly. Next, the membrane was gently shaken in 40 mL suspension of PBS-T20-dried milk powder (100:4 w/w%) for 30 minutes to block non-specific binding sites. Afterwards, the membrane was washed twice with PBS-T20 and finally soaked in 30 mL PBS-T20 containing 30 μL FITC-streptavidin. The mixture was gently shaken for 2 h in darkness and then the membrane was rinsed 3 times with 30 mL PBS-T20 to remove unbound streptavidin counterparts. The membrane was imaged using Bio-Rad ChemiDoc MR imaging system at 488 nm wavelength.

## Additional Information

**How to cite this article**: Lak, A. *et al*. Facile transformation of FeO/Fe_3_O_4_ core-shell nanocubes to Fe_3_O_4_ *via* magnetic stimulation. *Sci. Rep.* **6**, 33295; doi: 10.1038/srep33295 (2016).

## Supplementary Material

Supplementary Information

## Figures and Tables

**Table 1:** Magnetic, structural, and phase composition properties.

Notation	M_s_^298^/M_s_^5^	m	d_m_	T_b_	K_e_	H_c_	H_EB_	f_RS_/f_S_	a_RS_/a_S_	d_RS_(200)/d_S_(400)/(220)
initial	53/60	0.66	64	244	14.2	272	158	44/56	4.27/8.46	105/65/32
10 cycle	69/89	0.93	67	235	13.7	210	112	28/72	4.24/8.44	73/90/48
25 cycle	72/94	3.4	105	232	13.5	57	23	−/100	−/8.39	−/149/50
TA-130	79/92	4.3	117	226	13.1	20	5	—	−/8.35	−/155/61

*M*_*s*_, *m*, *d*_*m*_, *T*_*b*_, *K*_*e*_, *H*_*C*_, and *H*_*EB*_ obtained from the analysis of the magnetization curves and mass fraction *f*_*RS*_*/f*_*S*_, lattice constant *a*_*RS*_*/a*_*S*_ derived from the Rietveld analysis and crystallite sizes *d*_*RS*_*/d*_*S*_ of Fe_1−x_O (RS) and Fe_3−δ_O_4_ (S) estimated using the Scherrer formula. *M_s_* (emu/g_Fe_); *m* (A m^2^ × 10^–19^); *d_m_* (Å); *T_b_* (K); *K_e_* (kJ/m^3^); *H_C_* and *H_EB_* (mT); *f_RS_/f_S_* weight fraction (%); *a_RS_/a_S_* and *d_RS_/d_S_* (Å); TA-130: thermally annealed nanocubes at 130°C.
