# Supplementary material for: Facile transformation of FeO/Fe3O4 core-shell nanocubes to Fe3O4 via magnetic stimulation
Source: Sci Rep. 2016 Sep 26;6:33295. doi: 10.1038/srep33295 (PMC5036086; doi:10.1038/srep33295)
Supplement: Supplementary Information [file srep33295-s1.pdf]

Supplementary Information

Facile transformation of FeO/Fe_3O_4 core-shell nanocubes to Fe_3O_4 *via* magnetic stimulation

Aidin Lak,*[1,‡] Dina Niculaes,[1,‡] George C. Anyfantis,[1] Giovanni Bertoni,[2] Markus J. Barthel,[1] Sergio Marras,[1] Marco Cassani,[1] Simone Nitti,[1] Athanassia Athanassiou,[1] Cinzia Giannini[3] and Teresa Pellegrino*[1]

[1] *Istituto Italiano di Tecnologia, Via Morego 30, 16163 Genova, Italy.*

[2] *IMEM-CNR, Parco Area delle Scienze 37/A, 43124 Parma, Italy.*

[3] *Institute of Crystallography, National Research Council, via Amendola 122/O, Bari 70126 Italy.*

‡ *these authors have contributed equally to this manuscript*

E-mail: aidin.lak@iit.it, teresa.pellegrino@iit.it

Chemicals

Iron pentacarbonyl $Fe(CO)_5$ (98%), 1-octadecene (ODE, 99%), oleic acid (OLAC, 90%), triethylamine (99%), chloroform, ethanol (EtOH), dichloromethane (DCM), Dimethyl sulfoxide (DMSO), α,ω-aminopropyl-poly(ethylene glycol) (Mn=1500 g/mol), gallic acid, *N*-hydroxysuccinimide (NHS), *N*-(3-dimethylaminopropyl)-*N′*-ethylcarbodiimide hydrochloride (EDC), 2-(*N*-morpholino) ethanesulfonic acid hydrate (MES), phosphate buffered saline (PBS) (150 mM NaCl, pH 7.4), sodium chloride, sodium sulfate, sodium hydroxide, Tween20, NHS-activated biotin and fluorescein isothiocyanate (FITC) conjugated Streptavidin were purchased from Sigma-Aldrich. Sodium oleate (97%) were obtained from TCI. All the chemicals were utilized "as-received" and without further purification.

MES buffer was prepared at 0.6 M concentration and adjusted to pH 5.5 by titration with 1 M NaOH solution. For the ligand synthesis reaction, MES buffer solution was diluted to 0.1 M concentration. PBS-Tween20 (0.01 w%) (PBS-T20) buffer was made by dissolving 20 μL Tween-20 into 200 mL PBS (pH 7.4).

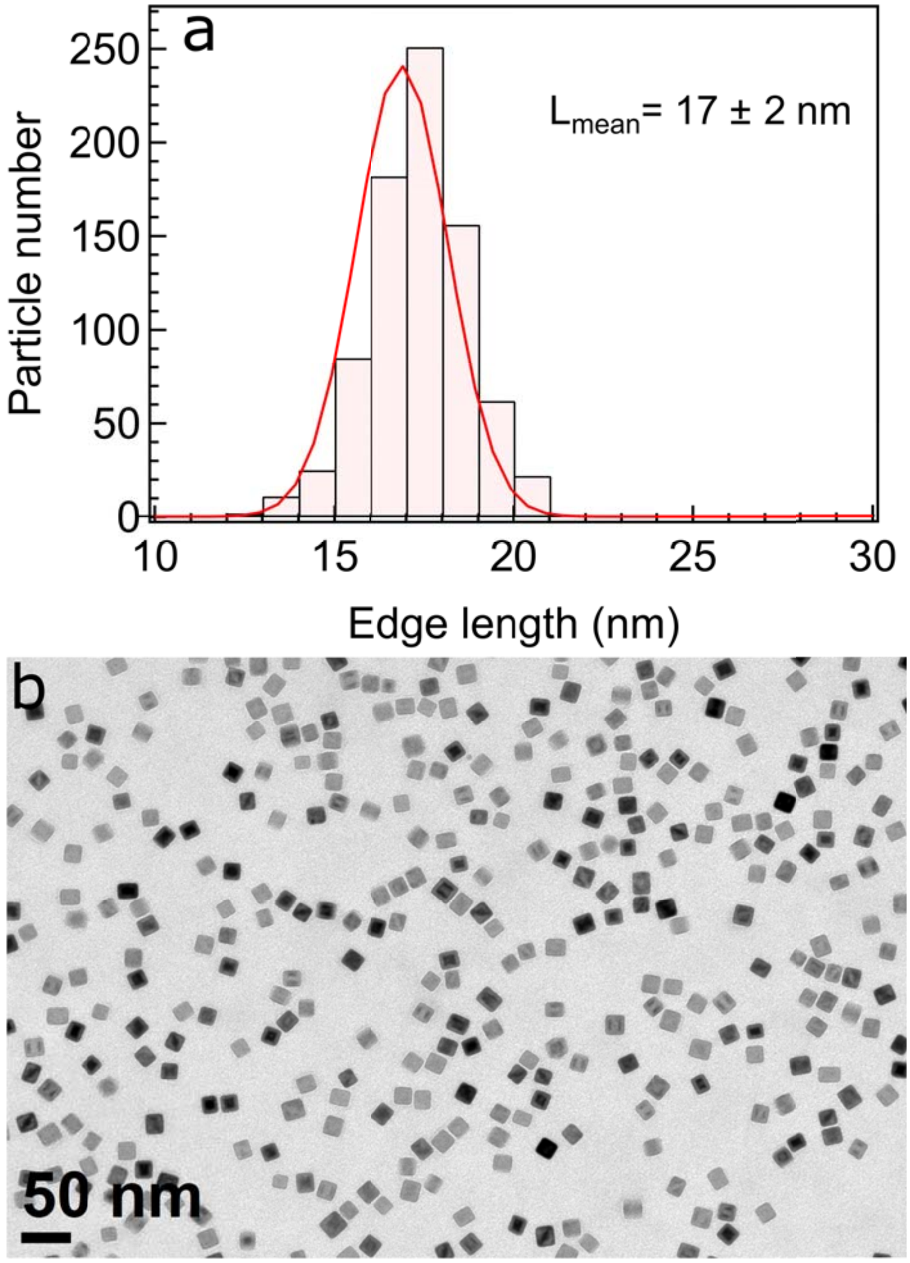

Figure S1. (a) Size histogram of as-synthesized nanocubes (shown in Fig. 1a, main paper) and (b) typical low resolution TEM image of the batch of nanocubes once coated with PEG (see below).

Post synthesis thermal annealing of nanocubes

Typically, in a 50 mL three neck flask, 20 mL particle suspension in chloroform containing 40 mg iron was added slowly to a mixture of 40 mL ODE and 2 mL OLAC at 70 °C in ambient atmosphere. The mixture was heated up from 70 to 130 °C in 20 minutes to slowly evaporate the chloroform and have the nanocubes well soluble in the organic mixture. The reaction mixture was then kept at this temperature for additional 5 h and within this period the solution color changes from black to dark brown. After cooling the reaction mixture to RT, the particles were isolated and washed following the procedure described in the previous section. The particles were dispersed in chloroform for further surface modification and characterization.

Gallic acid functionalization of α,ω-diaminopropyl poly(ethylene glycol) (Gallic-PEG-NH_2)

114 mg (0.67 mmol, 1 eq.) gallic acid was dissolved in a glass flask in 80 mL of MES/EtOH mixture (1:1 v%) and cooled down to 4 °C using ice bath. Subsequently, 257 mg (1.34 mmol, 2 eq.) EDC and 154 mg (1.34 mmol, 2 eq.) NHS were added and the solution was stirred for 30 min to activate the carboxyl group of the gallic acid. Afterwards, 1 g (0.67 mmol) α,ω-aminopropyl-poly(ethylene glycol) was added under vigorous stirring and the solution was warmed up to RT and stirred overnight. Next, the ethanol was removed under reduced pressure using rotary evaporator. Brine solution was added to the aqueous phase to facilitate the phase separation and the product was extracted three times with DCM. The combined organic phases were collected and dried with sodium sulfate. DCM was removed completely under reduced pressure. The final product was dried under vacuum at 40 °C.

Gallic-PEG-NH_2 polymer characterization by nuclear magnetic resonance spectroscopy (NMR)

^1H NMR spectroscopy was performed on a Bruker Ultra Shield Avance spectrometer (400 MHz). The NMR sample was prepared by dissolving 10 mg product in 0.8 mL of DMSO-*d6*.

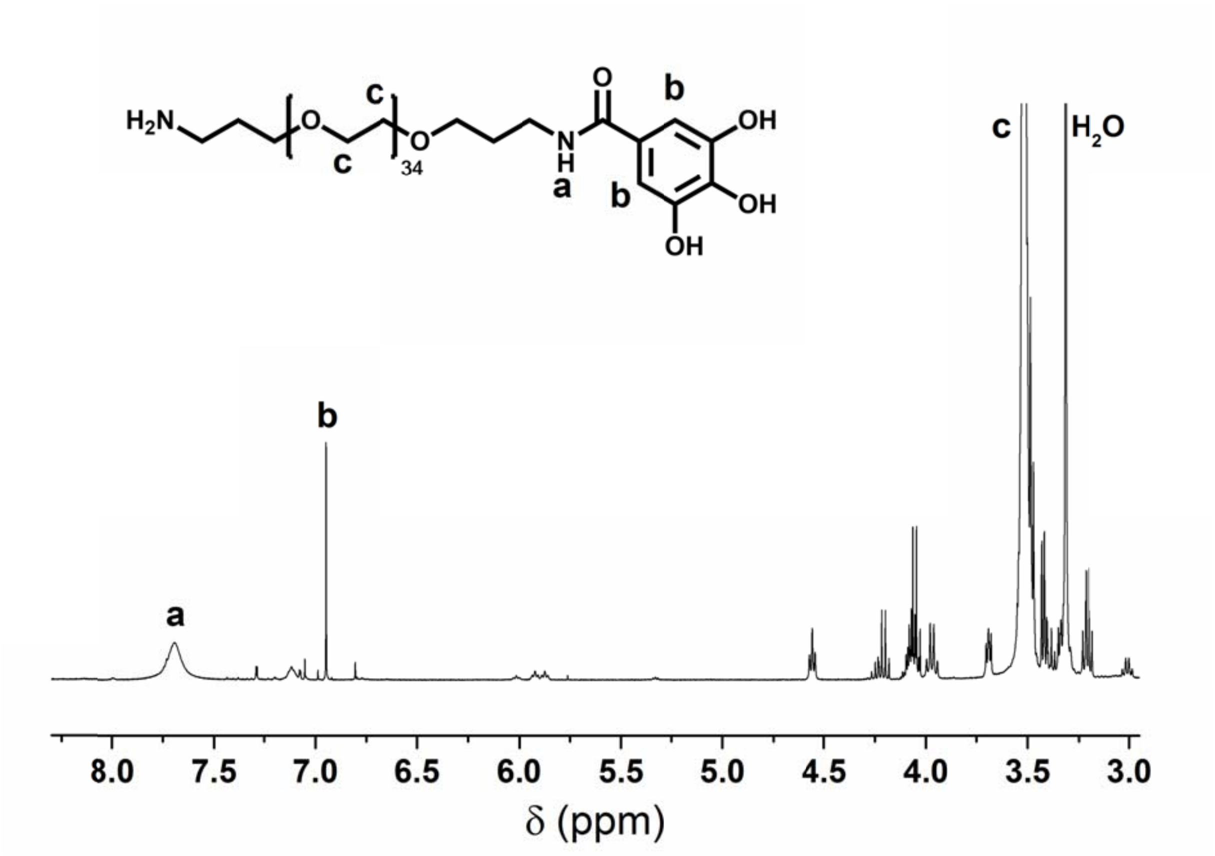

Figure S2. ^1H NMR spectrum of the synthesized α-gallic-ω-amino-poly(ethylene glycol) (Gallic-PEG-NH_2) measured in DMSO-*d6*.The degree of functionalization *f* = 30% was determined by integration of the ^1H signals of the amide bond (7.68 ppm, s, 1H, N-H), the aromatic protons (6.94 ppm, s, 2H, C-H) of the gallic acid and the PEG chain (3.63–3.43 ppm, m, 4H, CH_2-CH_2).

Geometric phase analysis of high resolution transmission electron microscopy images

The geometric phase analysis (GPA)[1] has been performed employing the FRWR tools plugin for DigitalMicrograph(TM) (Gatan, Inc.). In summary, GPA measures the relative displacement of lattice fringes compared to a reference area from a HRTEM image to calculate the strain tensor (ε_{ij}) and the rotation (ω_{xy}). In GPA analysis, the Fourier component **g** of a particular set of fringes are selected. The intensity of these fringes is given by

$$I_g(\boldsymbol{r}) = A_g(\boldsymbol{r})\exp(iP_g(\boldsymbol{r}))$$

with $A_g(\mathbf{r})$ the amplitude and $P_g(\mathbf{r})$ the phase.

The relative phase shift induced by a small displacement of the fringes **u**(**r**) with respect to the selected reference region is given by

$$P_g(\boldsymbol{r}) = -2\pi\boldsymbol{g}.\boldsymbol{u}(\boldsymbol{r})$$

By taking two non-collinear **g** components into analysis, the two-dimensional displacement field holds

$$\boldsymbol{u}(\boldsymbol{r}) = \frac{-1}{2\pi}(P_{g1}(\boldsymbol{r})\boldsymbol{a_1} + P_{g2}(\boldsymbol{r})\boldsymbol{a_2})$$

with $\mathbf{a_1}$ and $\mathbf{a_2}$ lattice vectors corresponding to $\mathbf{g_1}$ and $\mathbf{g_2}$ in the real space. The strain tensor and rotation are defined by[2]

$$\varepsilon_{ij} = \frac{1}{2}(\frac{\partial u_i}{\partial x_j} + \frac{\partial u_j}{\partial x_i})$$

$$\omega_{xy} = \frac{1}{2}(\frac{\partial u_y}{\partial x} + \frac{\partial u_x}{\partial y})$$

Here, in a typical GPA analysis, a Fourier filtered power spectrum of an HRTEM image is used to generate amplitude and phase shift images. The phase shift of the lattice fringes relative to a reference region (with phase fluctuations < ± 0.2) is employed to build the corresponding strain (ε_{ij}) and rotation (ω_{xy}) maps.

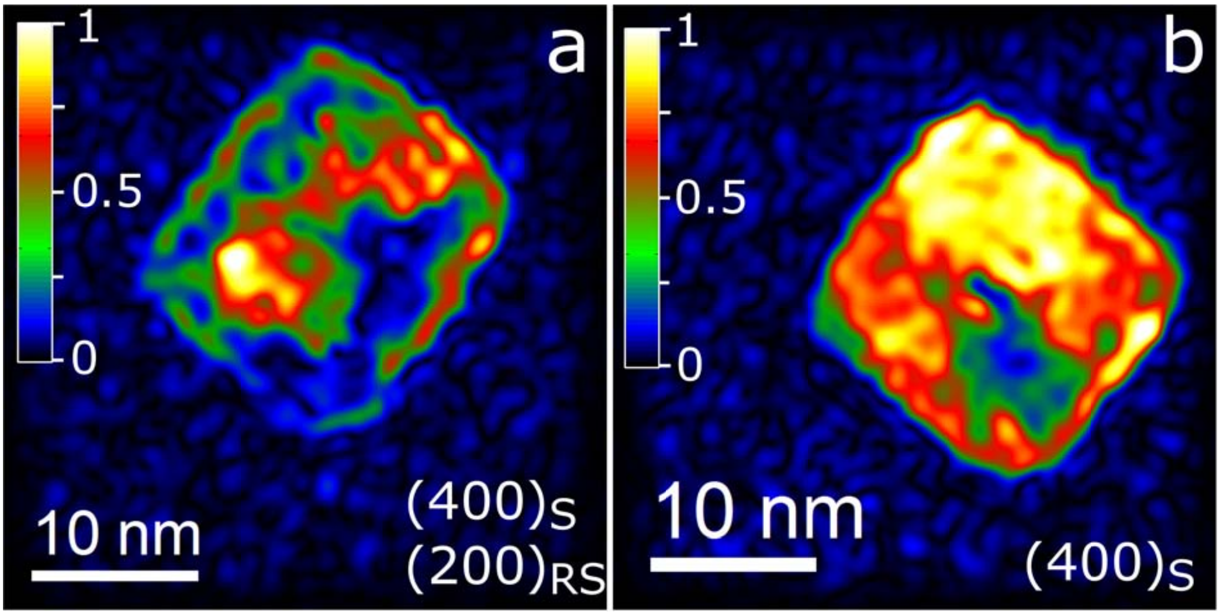

Figure S3. Amplitude map of relative intensities of (a) $\{400\}_S$-$\{200\}_{RS}$ fringes of initial core-shell and (b) $\{400\}_S$ fringe of 25 MH cycles treated nanocubes, respectively, acquired from the GPA analysis. S and RS sub-indices stand for Fe_3O_4 inverse spin (S) and FeO rock-salt (RS) crystal structure, respectively.

Since the d-spacing of $(200)_{RS}$ and $(400)_S$ planes are very close, the amplitude map of the initial nanocubes (Fig. S3a), obtained from GPA analysis, comprises of the contribution of both fringes. The higher relative intensity at the center of the initial particle indicates the presence of well crystalline FeO phase. Oppositely, a significantly lower relative intensity at the shell of the

particles implies that the $(400)_S$ planes ascribed to magnetite are not well ordered and this phase is rather defected.

On the other hand, in the amplitude map of the treated nanocubes (Fig. S3b), a rather uniform relative intensity of $(400)_S$ plane is seen. The GPA analysis on HRTEM micrograph was conducted by taking the contribution of $(400)_S$ fringe into account. Observing such high intensity for this plane without taking FeO fringe into analysis means that there is a significant occurrence of the ordered $(400)_S$ planes throughout the whole particle. This is an indication of the formation of ordered magnetite phase in the treated particles.

Analysis of the magnetization curves

To derive magnetic moment distribution from M-H curves, the reconstructions of M-H curves were performed using MINORIM non-regularized inversion method using a discrete form of the Langevin function given by

$$M(H_j) = \sum_{0}^{\infty} m_i L(m_i, H_j) n_i$$

$$L(m_i, H_j) = \coth\left(\frac{\mu_0 m_i H_j}{k_B T}\right) - \frac{k_B T}{\mu_0 m_i H_j}$$

where m_i is the mean magnetic moment of ith size bin with an amplitude equals to n_i, μ_0 is the permeability of vacuum and k_B is the Boltzmann constant.[3]

This approach assumes no specific shape and number of modality for the distribution function, being advantageous for the accurate determination of magnetic moment distribution from M-H curves. The M_s and the mean magnetic moment m were set as fit parameters.

To obtain the size of magnetic domain d_m from the m, firstly, the M_s values given in emu/g_{Fe} were converted to SI unit in A/m using

$$M_s(A/m) = M_s(emu/g_{Fe}) \times \rho(g/cm^3) \times 1000$$

For the core-shell nanocubes, the effective density ρ_e was calculated using

$$\rho_e = \rho_{FeO} \times f_{FeO} + \rho_{Fe_3O_4} \times f_{Fe_3O_4}$$

with f_{FeO} and f_{Fe3O4} the volume fraction of $Fe_{1-x}O$ and $Fe_{3-\delta}O_4$ phases extracted from the XRD Rietveld analysis and ρ_{FeO} (6.17 g/cm^3) and $\rho_{Fe_3O_4}$ (5.14 g/cm^3). Eventually, the magnetic size d_m holds

$$d_m(nm) = \sqrt[3]{\frac{m}{M_s}}$$

The exchange H_E and coercive H_C fields can be written as

$$H_E = \frac{-(H^+ + H^-)}{2}$$

$$H_c = \frac{(H^+ - H^-)}{2}$$

in which H^+ and H^- are the positive and negative coercivities taken from the FC hysteresis loops.

The magnetocrystalline anisotropy constant (K_e) was calculated using the Néel-Brownian relaxation equation as described by

$$K_eV = k_BT_B\ln(\frac{\tau_m}{\tau_0})$$

with the inverse attempt frequency τ_0=10^{-9} s, the measurement time τ_m≈1 s and the particle volume V_c=17^3 nm^3. The superparamagnetic blocking temperatures T_b were estimated from the maximum in ZFC curve and data are summarized in table 1 in the main paper.

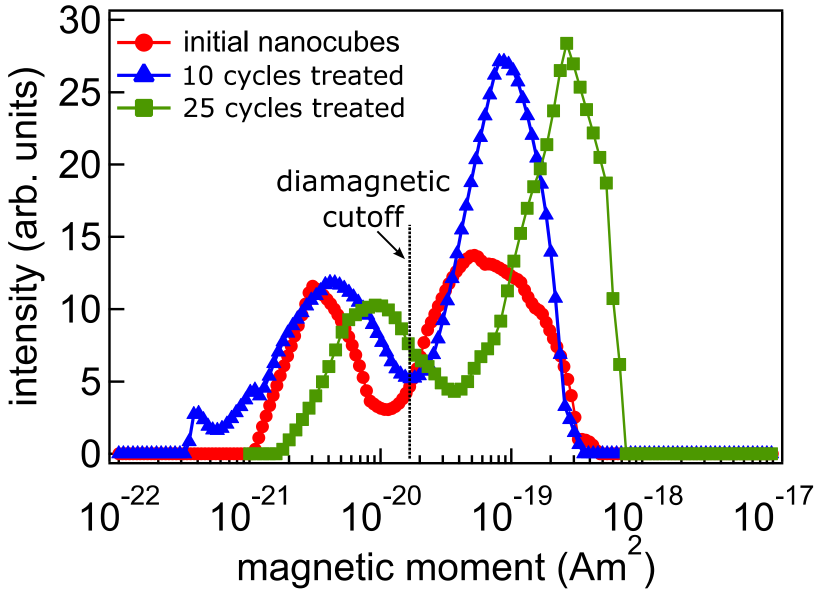

Figure S4. Magnetic moment distributions of initial, 10 and 25 MH cycles treated nanocubes derived from the fits to the room temperature M-H curves. The peak appeared around 10^{-21}-10^{-20} Am^2 is below the diamagnetic cutoff chosen for the fits and thus physically meaningless.

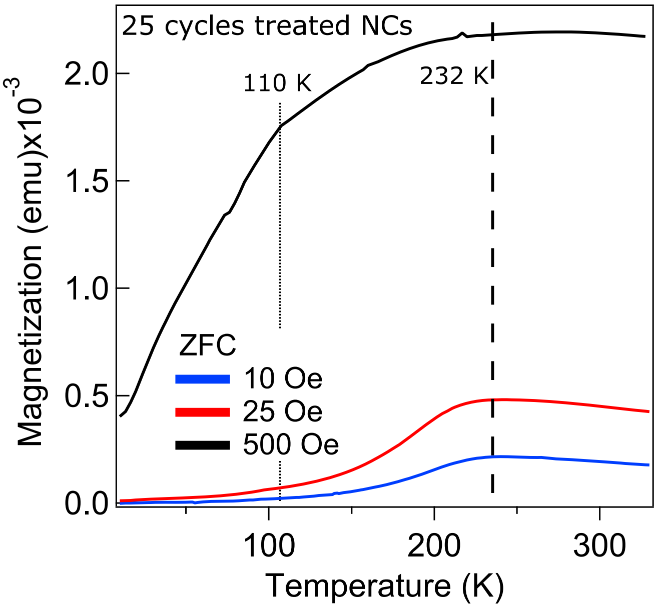

Figure S5. Temperature dependent ZFC magnetizations measured at 1, 2.5 and 50 mT on 25 MH cycles treated nanocubes. The results show that the ZFC maxima (corresponding to the blocking temperature) is independent of the applied field and remains at 232 K.

Quantitative Rietveld crystal structure analysis

In this study, the Rietveld crystal structure method has been exploited to simulate the experimental XRD patterns, monitor temporal evolution of particle phase composition and lattice constant. The refinements were carried out using FullProf Suite program.[4] Firstly, the instrumental resolution function (IRF) file was generated by performing the profile matching on the LaB_6 NIST SRM 660a standard sample XRD pattern. The IRF file was uploaded into the program in order to take into account the instrumental broadening. The Thompson-Cox-Hastings Pseudo-Voigt with an axial divergence asymmetry was selected as a peak shape. The background

was set to a linear interpolation of background points obtained using the WinPLOTR software (FullProf package). The free parameters which have shown more than 50% cross correlation were refined independently. The refined parameters were limited to scale factor, lattice constants and the first two parameters of anisotropic Lorentzian size broadening. The simulated patterns are shown in Fig. S6.

To estimate the crystallite size, the Scherrer formula as written

$$d = \frac{k\lambda}{\beta \cos(\theta)}$$

with k_{220} = 0.789 and k_{200} = 0.862 the anisotropy constants[5], λ = 0.1540 nm the wavelength of Cu-K_{α}, β the full maximum at half width (FWHM) and θ the Bragg angle in radians, was used. The peak shape was assumed to be Gaussian. The overlapping reflections such as $(200)_{RS}$ and $(400)_{S}$ at θ = 42° and $(220)_{RS}$ and$(440)_{S}$ θ = 62° were deconvoluted by superposition of two Gaussian peaks. An example of the double peak fit is demonstrated in Fig. S5d for the initial nanocubes.

Table S1. Atom positions, sites and occupancies used in the Rietveld analysis of initial, 10 and 25 cycles MH treated nanocubes for both $Fe_{1-x}O$ and $Fe_{3-\delta}O_4$ phases.

$Fe_{3-\delta}O_4$						
atom	valence	x	y	z	site	occupancy
Fe(t)	2+	0.125	0.125	0.125	8a	0.375
Fe(o)	3+	0.5	0.5	0.5	16d	0.75
O	2-	0.25	0.25	0.25	32e	1.5
$Fe_{1-x}O$						
Fe	2+	0	0	0	4a	1
O	2-	0.5	0.5	0.5	4b	1

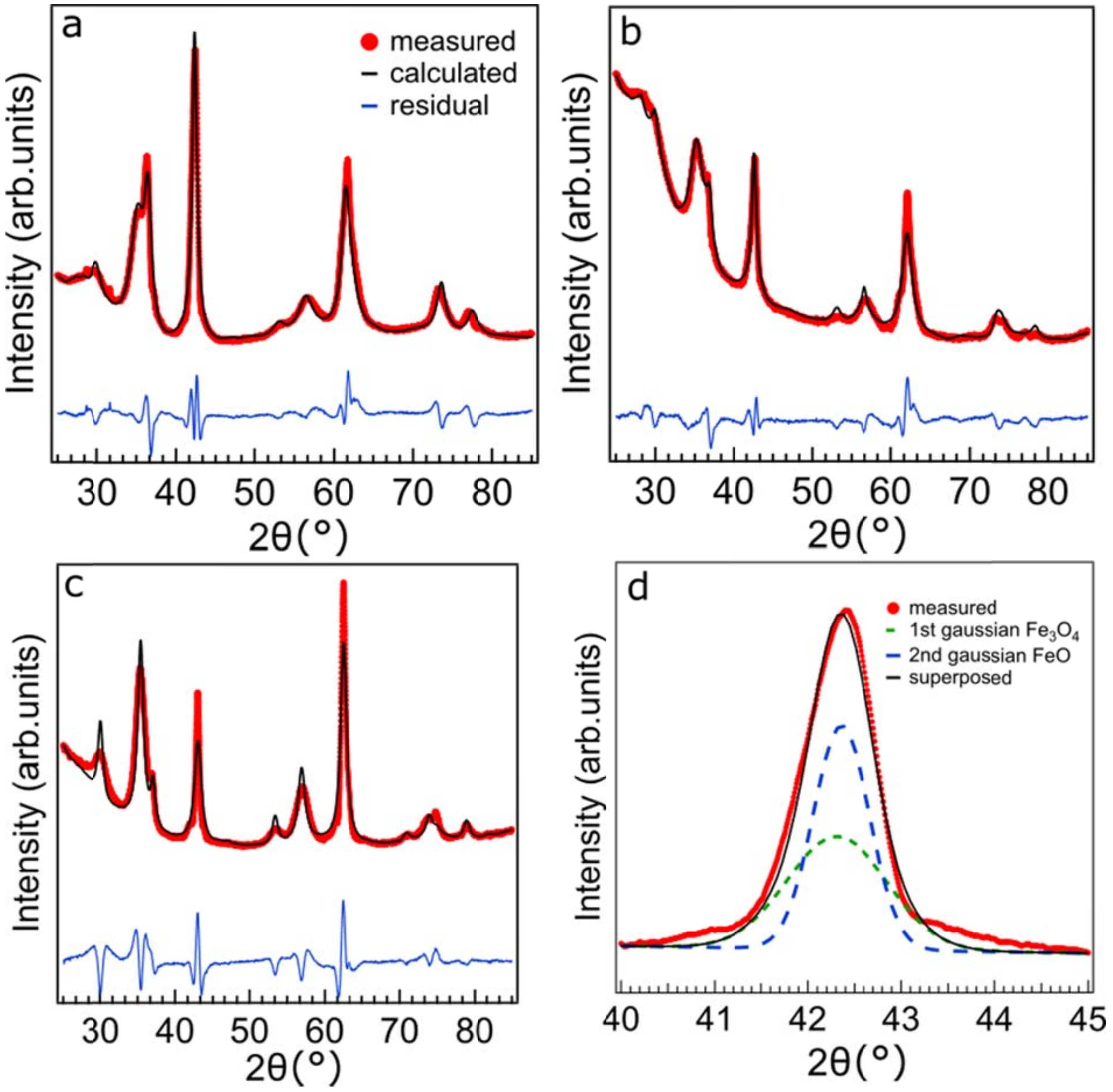

Figure S6. XRD pattern (red symbols) and corresponding Rietveld refinement (solid black lines) of (a) initial core-shell nanocubes, (b) 10 and (c) 25 MH cycles treated nanocubes (fit residuals are shown as blue lines), and (d) deconvolution of overlapping peaks by superposition of two Gaussian peaks as here shown for $(200)_{RS}$ and $(400)_{S}$ reflections at $\theta = 42°$.

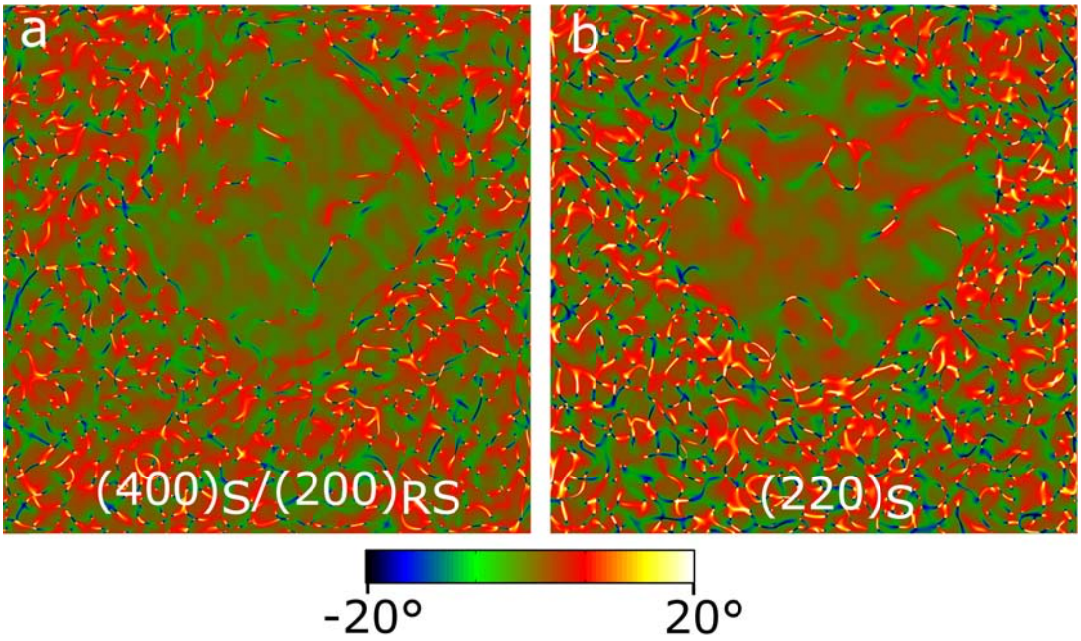

Figure S7. Rotational deformation ω_{xy} map of (a) $\{400\}_S/\{200\}_{RS}$ and (b) $\{220\}_S$ fringe of initial core-shell nanocubes.

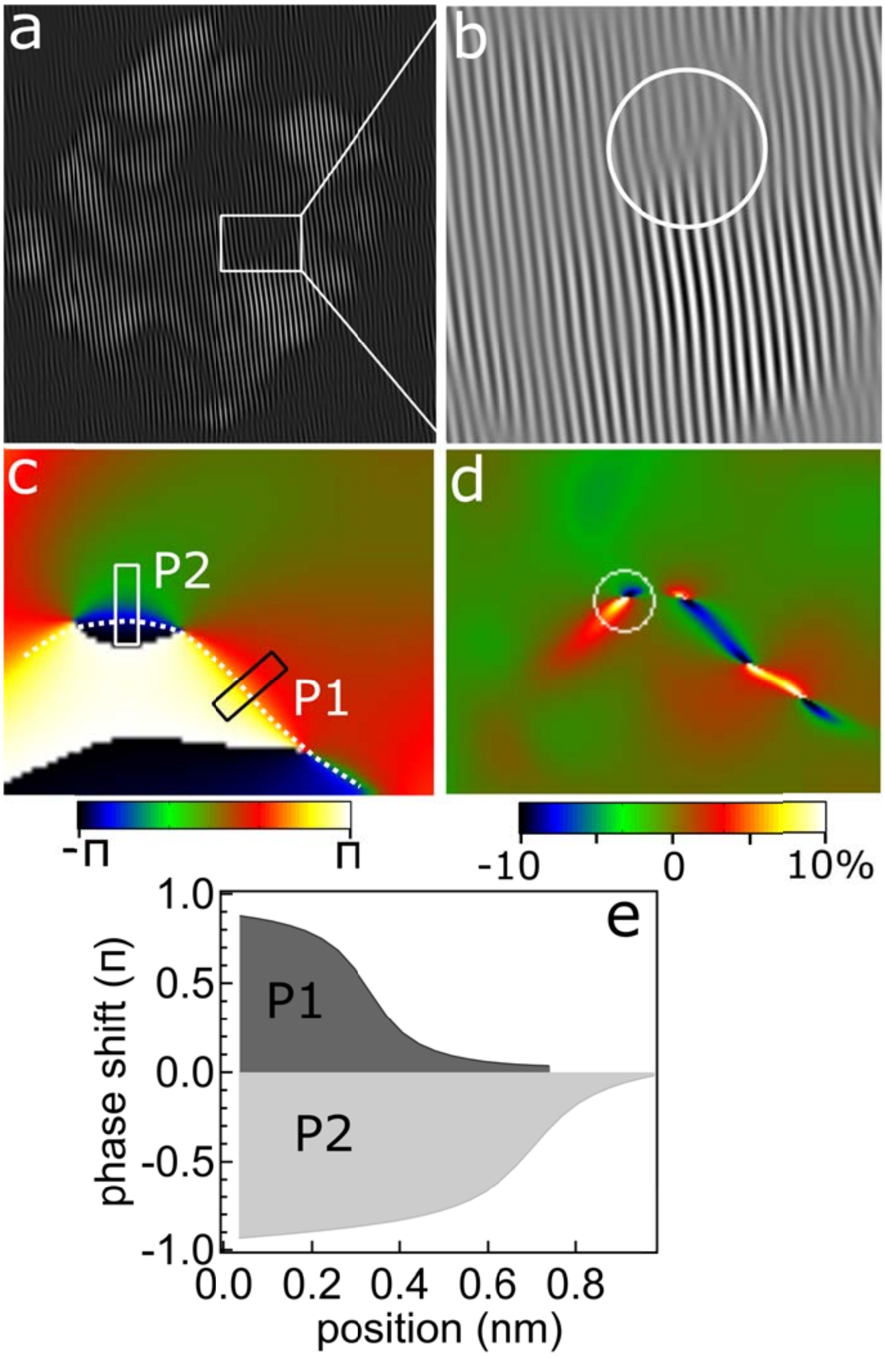

Figure S8. (a) Fourier filtered image of initial nanocubes obtained by masking $\{220\}_S$ spinel fringe, (b) lattice fringe image, (c) the corresponding phase and (d) lattice deformation ε_{xy} obtained by GPA analysis and (e) relative phase shift profiles along two APBs denoted by P1 and P2 in the phase map.

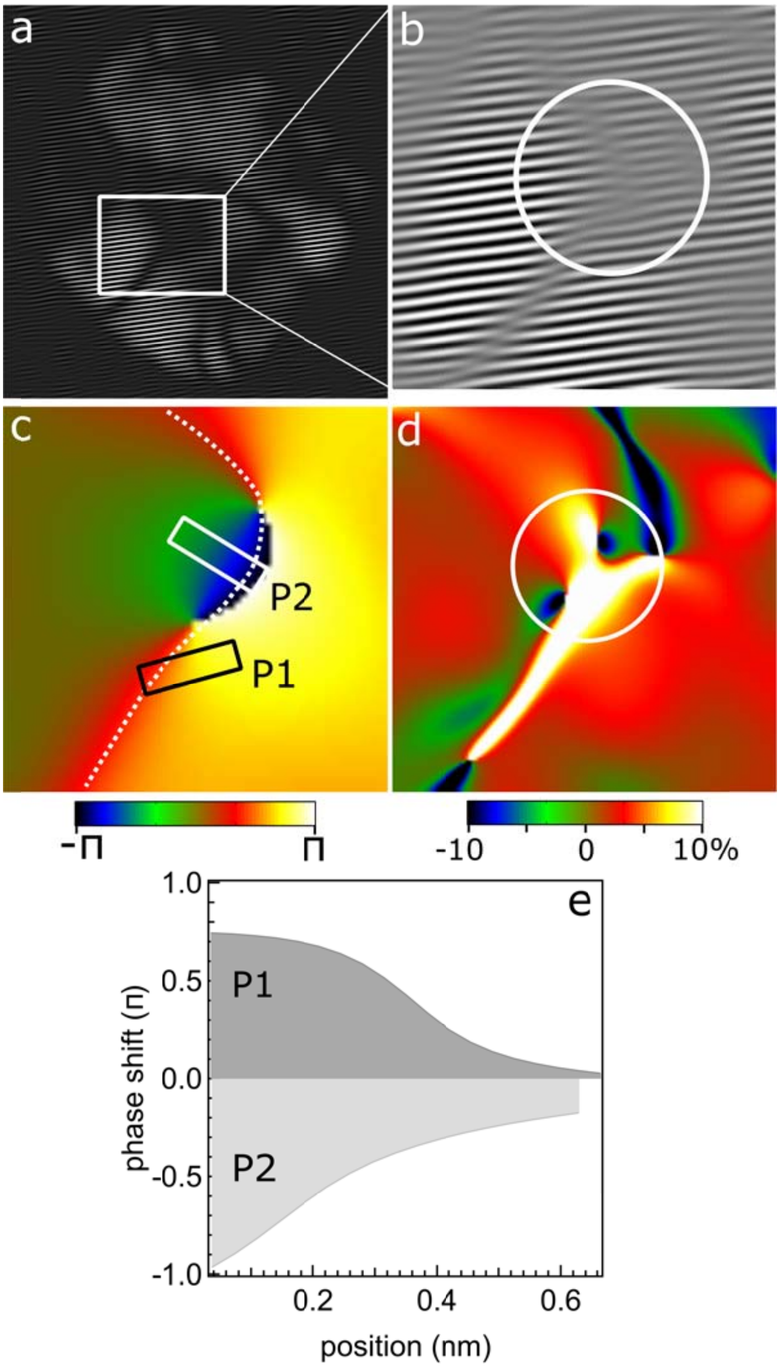

Figure S9. (a) Fourier filtered image of 25 MH cycles treated nanocubes obtained by masking $\{220\}_S$ spinel fringe, (b) lattice fringe image, (c) the corresponding phase and (d) lattice deformation ε_{xy} obtained by GPA analysis and (e) relative phase shift profiles along two APBs denoted by P1 and P2 in the phase map.

The antiphase boundaries (APBs) at which the neighbor Fe_3O_4 subdomains are shifted by ¼ a_0[110] can be identified by ± π shift in the phase map from the $(220)_S$ fringe.[3] For the initial nanocubes, the phase and strain ε_{xy} maps are shown in Fig. S8c,d. Along two areas denoted by P1 and P2, a discontinuity in both phase and deformation maps can be observed. And, the phase shifts from +π/-π to 0, respectively, within ≈ 1 nm length (Fig. S8e). The APBs can be seen in the Fourier filtered lattice fringe image acquired by masking the $(220)_S$ spot as highlighted in Fig. S8b. By performing the same GPA analysis on the 25 MH cycles treated nanocubes (see Fig. S9), it has been found that the APBs do persist although the Fe_3O_4 domain size grows nearly twice. Indeed, the APBs separate the Fe_3O_4 domains, accounting for the retarded growth of the size of the $(220)_S$ diffraction line (Table 1, main paper).

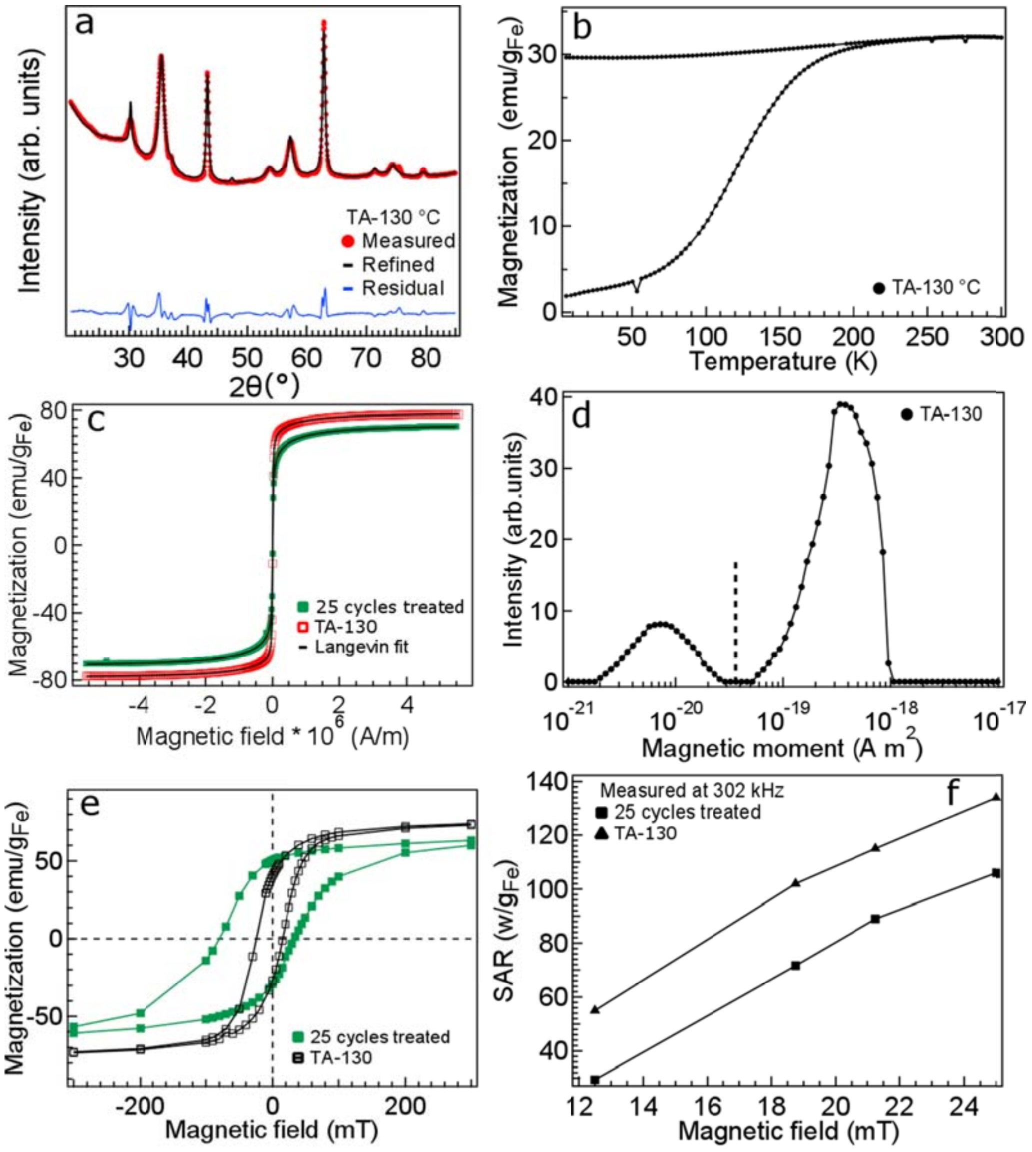

Figure S10. Structural and magnetic characterization of nanocubes thermally annealed at 130 °C TA-130. (a) XRD pattern (red symbols) and Rietveld refinement (black lines), (b) ZFC and FC temperature dependent magnetizations recorded in 10 mT, (c) magnetization hysteresis loops recorded at 298 K, (d) magnetic moment distribution obtained from the analysis of the room temperature magnetization curve (a peak appeared below the dashed line is physically meaningless), (e) FC hysteresis loops of particles cooled to 5 k in 5 T, (f) SAR as a function of the magnetic field measured at 302 kHz.

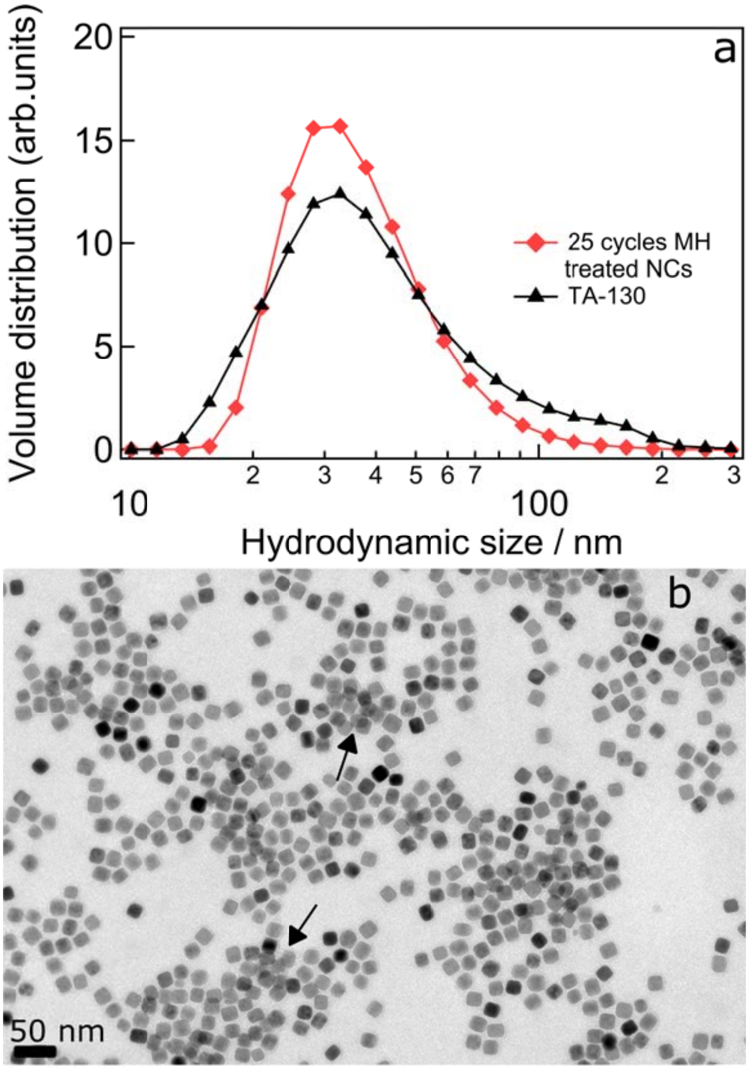

Figure S11. (a) Volume weighted hydrodynamic size distribution and (b) typical low resolution TEM micrograph of water dispersible PEG coated thermally annealed (TA-130) nanocubes. The hydrodynamic size distribution of 25 cycles magnetically oxidized nanocubes is plotted for comparison.

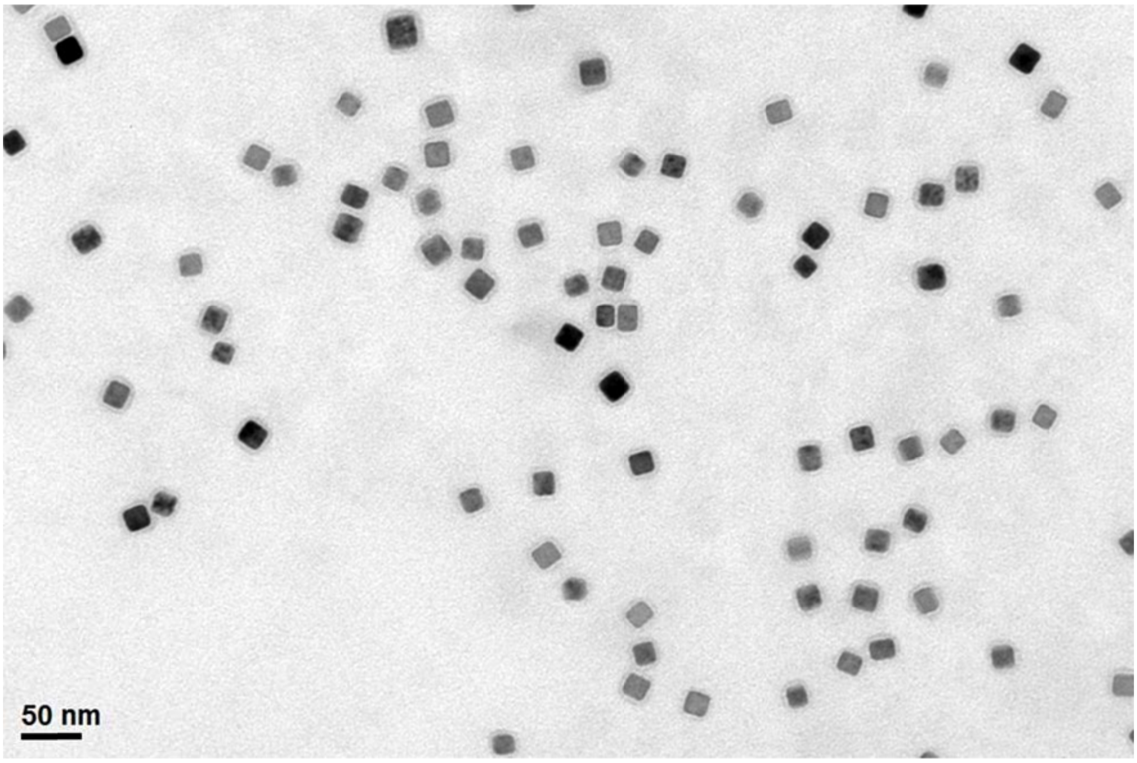

Figure S12. Survey TEM micrograph of 25 MH cycles treated PEGylated nanocubes. It can clearly be seen that the shape and the single-core nature of the nanocubes remain intact after applying a full MH treatment.

References

[1] Hytch, M. J.; Snoeck, E.; Kilaas, R. Ultramicroscopy 1998, 74, 131-146.

[2] Tai, C. W.; Lereah, Y. Appl. Phys. Lett. 2009, 94, 051908.

[3] Rijssel, J. V.; Kuipers, B. W.M.; Erné, B. H. J. Magn. Magn. Mater. 2014, 353, 110–115.

[4] Rodriguez-Carvajal, J. FullProf Suite; LLB Saclay & LCSIM: Rennes, France, 2003.

[5] Li, Z. Part. Syst. Charact. 2011, 28, 19-24.
